# Evaluation of Antimicrobial Activity, Toxicity, and Stability of *Andrographis paniculata* Intimate Wash Formulation

**DOI:** 10.4014/jmb.2602.02037

**Published:** 2026-09-17

**Authors:** Nur Rina Alissa Razian, Anis Amiera Muhamad Alojid, Khong Ling Huey, Tuan Nadrah Naim Tuan Ismail, Tuan Noorkorina Tuan Kub

**Affiliations:** 1 Department of Medical Microbiology and Parasitology, School of Medical Sciences, Universiti Sains Malaysia, 16150 Kubang Kerian, Kelantan, Malaysia; 2 School of Dental Sciences, Universiti Sains Malaysia, 16150 Kubang Kerian, Kelantan, Malaysia; 3 Medical Microbiology and Parasitology Laboratory, Hospital Pakar Universiti Sains Malaysia, 16150 Kubang Kerian, Kelantan, Malaysia

**Keywords:** *Andrographis paniculata*, Antimicrobial, Intimate wash, Stability, Toxicity, Vaginal pathogen

## Abstract

Vaginal infections are commonly treated with antibiotics but increasing antimicrobial resistance and side effects of commercial intimate wash have encouraged the search for safer herbal alternatives. This study aimed to develop and evaluate an intimate wash formulated with *Andrographis paniculata* extract against selected pathogens; *Candida albicans*, *Neisseria gonorrhoeae*, *Escherichia coli* and *Streptococcus agalactiae*. The antimicrobial activity of *A. paniculata* intimate wash was assessed by using the agar well diffusion method to observe inhibition zones. Toxicity evaluations were conducted, including heavy metal detection for the intimate wash and the brine shrimp lethality assay for the extract. Stability and sterility testing of intimate wash were also conducted to determine the product’s shelf-life. The results showed that the intimate wash exhibited optimum inhibition at 3.125 mg/mL. Heavy metals were below the permissible limits, and the extract also demonstrated non-cytotoxic effect towards brine shrimp. The intimate wash showed good stability and remained free from contamination throughout 24 months of storage. In conclusion, *A. paniculata* intimate wash is acceptable for preliminary safety findings and has potential as herbal alternative for the management of vaginal infections. However, further *in vivo* and clinical studies are required to confirm its safety and efficacy.

## Introduction

Vaginal health issues are among the most common gynaecological conditions in women of reproductive age and represent a major cause of morbidity worldwide. These conditions often present with symptoms such as itching, foul-smelling odour [1], and excessive vaginal discharge [2]. Poor hygiene practices such as improper menstrual hygiene and unprotected sexual intercourse can disrupt the vaginal microbiome [3]. This condition often predisposes women to various vaginal infections [4]. *Candida albicans* is a common normal vaginal flora in reproductive-age women. Usually, it exists as harmless commensals within the vaginal microbiota. However, some factors can trigger its transition into opportunistic pathogens [5].

Gonorrhoea, which is caused by *Neisseria gonorrhoeae*, is one of the most serious sexually transmitted infections (STIs) and can also lead to neonatal conjunctivitis if transmitted during childbirth [6]. Several behavioural and social factors can contribute to STI transmission [7]. These infections can link to various severe complications [8, 9]. In addition, urinary tract infection (UTI) is commonly caused by uropathogenic *Escherichia coli* [10]. Due to poor hygiene, its colonization can adhere and invade the vaginal epithelial cells [11]. Besides, *Streptococcus agalactiae* commonly colonizes the human gastrointestinal and female genitourinary tracts asymptomatically. Although it is best known for causing invasive infections in neonates, it is also associated with disease in pregnant women, the elderly, and immunocompromised individuals [12].

The World Health Organization (WHO) recognizes antimicrobial resistance when microorganisms develop reduced susceptibility to antimicrobial agents, and the overuse of antibiotics in recurrent vaginal infections can accelerate resistance development [13]. In addition, some commercial intimate washes contain harmful ingredients such as sodium lauryl sulphate (SLS) and parabens, raising safety concerns about irritation and long-term reproductive health risks [14, 15]. These concerns emphasize the importance of developing intimate wash formulations that are both effective against pathogens and safe for the vaginal microbial ecosystem. In response, many women are turning to herbal-based alternative treatments. In recent years, the use of herbal intimate washes has gained increasing popularity among women as part of their daily personal care routine [16].

Herbal-based formulations are perceived as safer and more natural options for maintaining intimate hygiene, as they are believed to produce fewer side effects compared to chemical-based commercial products [17]. However, despite their widespread use, there remains a lack of scientific evidence and standardized guidelines concerning the safety and antimicrobial efficacy of intimate wash, specifically against the vaginal pathogens [18]. A finding from previous study by Srivastava *et al*. [19] demonstrated the antimicrobial activity in soap formulation, showing effectiveness against *Escherichia coli*. Another study by Razian *et al*. [20] also reported that *A. paniculata* preserves the growth of beneficial *Lactobacillus crispatus*, which outperforms several commercial intimate washes in Malaysia.

In this context, *A. paniculata* is considered as one of the most promising herbal candidates for alternative treatments against microbial infections. A study reported that the methanolic leaf extract of *A. paniculata* exhibited strong antimicrobial activity against *E. coli*, with a minimum bactericidal concentration of 3.31 mg/mL [21]. Similarly, a study also reported that *A. paniculata* demonstrated stronger antimicrobial effects against *C. albicans* compared to *Azadirachta indica* and *Curcuma longa* [22]. Toxicological evaluation also exhibited inhibitory effects on human breast cancer cell lines while maintaining the viability of normal fibroblast cells [23], suggesting low cytotoxicity and potential safety for topical use.

Therefore, this study aims to address this research gap by developing an intimate wash formulated with *A. paniculata* extract that demonstrates effective antimicrobial activity against key vaginal pathogens while ensuring product safety through the exclusion of harmful ingredients [24]. Toxicity evaluations involving heavy metal detection in intimate wash and cytotoxicity testing using brine shrimp lethality assay for the extract were also conducted to assess its suitability for intimate use. Additionally, stability and sterility testing of *A. paniculata* intimate wash were included to ensure the product’s long-term integrity and microbiological safety [25, 26]. The findings highlight *A. paniculata* intimate wash as a promising, sustainable alternative to conventional chemical products, demonstrating both antimicrobial efficacy and compatibility with the vaginal microbiota.

## Materials and Methods

### Preparation of the *A. paniculata* Extract

The extract was prepared using the maceration method [27]. Briefly, 100 g of *A. paniculata* leaves powder was soaked in 1 L of absolute ethanol and maintained at 30°C overnight on a C-MAG HS 7 hot plate stirrer (IKA, India). The mixture was subsequently filtered using Whatman^TM^ sterile filter paper (CAT No. 1001−150; Merck KGaA, Germany), and the remaining solids on the filter paper were discarded. The filtrate was concentrated to a paste using a rotary evaporator (BÜCHI Labortechnik AG, Switzerland). After the paste was dried in laminar flow for 24 h, it was stored at 2 to 5°C for further use.

### Formulation of *A. paniculata* Intimate Wash

The paste obtained from the extract was subsequently weighed to formulate *A. paniculata* intimate wash at five different concentrations: 50, 25, 12.5, 6.25 and 3.125 mg/mL. This formulation was adapted from a previously reported formulation [20]. Initially, the paste was dissolved in 1% dimethyl sulfoxide (DMSO) and combined with 2% of fragrance agent (peppermint oil), solubilizing agent (Tween 20), preservatives (potassium sorbate and sodium benzoate), surfactants (sodium coco-glucoside tartrate, cocamidopropyl betaine, and sodium cocoyl hydrolyzed wheat protein), and sterile deionized water as the solvent. Citric acid was added to adjust the final pH to 4.0. All preparation steps were conducted under aseptic conditions to maintain sterility.

### Bacterial and Fungal Stock Cultures

*Candida albicans* (ATCC^®^90028^TM^), *Neisseria gonorrhoeae* (ATCC^®^49226^TM^), and *Escherichia coli* (ATCC^®^25922^TM^) were obtained from the American Type Culture Collection (ATCC, USA) as standardized reference strains. In contrast, *Streptococcus agalactiae* (Strain ID: 23041118) was obtained from the Microbiology Laboratory Stock Culture at Universiti Sains Malaysia, Kubang Kerian, Kelantan, Malaysia, and was confirmed using the VITEK^®^2 automated identification system (bioMérieux, Inc., France), achieving an accuracy exceeding 98%. All strains were preserved under appropriate storage conditions within the departmental microbial culture repository for future research use.

For subculturing, *E. coli* and *S. agalactiae* were grown on Columbia Sheep Blood Agar and incubated aerobically for 18−24 h at 37°C. *C. albicans* was subcultured on Sabouraud Dextrose Agar and incubated aerobically for 24 h at 37°C, while *N. gonorrhoeae* was cultured on Chocolate Horse Blood Agar and incubated anaerobically for 48 h at 37°C under the appropriate atmospheric conditions.

For antimicrobial study, Mueller Hinton Agar was used to grow *E. coli* and incubated for 18 h at 37°C. Potato Dextrose Agar was used to grow *C. albicans*, while *S. agalactiae* was grown on Mueller Hinton Blood Agar and incubated for 24 h at 37°C. *N. gonorrhoeae* was grown on Mueller Hinton Blood Agar and incubated anaerobically for 48 h at 37°C under the appropriate atmospheric conditions.

### Neutralization Validation Assay

To evaluate whether residual antimicrobial activity of the *A. paniculata* intimate wash formulation could interfere with microbial recovery during sterility assessment, a neutralization validation assay was performed prior to sterility testing. Neutralization was validated for each formulation concentration (0.1%, 0.5%, 1.0%, and 5.0%, v/v) used in the sterility test to confirm the effectiveness of Dey-Engley Neutralizing Broth under the test conditions [28].

Briefly, each concentration of the intimate wash formulation containing *A. paniculata* extract, potassium sorbate, sodium benzoate, and citric acid was mixed with an equal volume of Dey-Engley Neutralizing Broth. The mixtures were vortexed thoroughly and allowed to stand at room temperature for 10 min to facilitate neutralization of residual antimicrobial activity.

Neutralization efficacy and neutralizer toxicity were evaluated using *E. coli* as a representative Gram-negative organism. *E. coli* was selected because of its well-established use in antimicrobial neutralization studies, reproducible growth characteristics, and sensitivity to residual antimicrobial agents, making it a suitable indicator for detecting residual antimicrobial carry-over. Standardized microbial suspensions equivalent to approximately 1 × 10^6^ colony-forming units (CFU) per mL were prepared.

For each formulation concentration, four experimental groups were established: (i) a microbial suspension only, serving as the viability control; (ii) a microbial suspension with Dey-Engley Neutralizing Broth, serving as the neutralizer toxicity control; (iii) a microbial suspension exposed to the formulation without neutralization, serving as the antimicrobial activity control; and (iv) a microbial suspension exposed to the formulation after neutralization with Dey-Engley Neutralizing Broth, serving as the neutralization validation group.

Following the 10-min neutralization period, the standardized *E. coli* suspension was added to the neutralized formulation in the neutralization validation group. Aliquots from all experimental groups were then serially diluted in sterile saline and plated onto appropriate agar media. The plates were incubated at 37°C for 18–24 h, after which microbial recovery was assessed by qualitative observation of colony growth. Neutralization was considered effective when microbial recovery in the neutralization validation group was comparable to that of the viability control, while the neutralizer toxicity control demonstrated no inhibitory effect on microbial growth and the antimicrobial activity control exhibited reduced or no microbial recovery.

### Agar Well Diffusion Assay for *A. paniculata* Intimate Wash

Wells with a diameter of 6 mm were punched at equal intervals across the agar plate using a cork borer. Freshly subcultured isolates were suspended in 3 mL of Mueller Hinton Broth for *N. gonorrhoeae*, *E. coli*, and *S. agalactiae*, and in RPMI 1640 medium for *C. albicans*, and adjusted to 0.5 McFarland turbidity standard. Then, a sterile swab containing the suspension was used to spread onto the agar surface. Each well was then filled with 60 μL of *A. paniculata* intimate wash at concentrations of 50, 25, 12.5, 6.25, and 3.125 mg/mL, along with negative control (NC) of 60 μL sterile distilled water. The corresponding volume of SebaMed^®^ Feminine Wash was used as positive control (PC). *C. albicans*, *E. coli*, and *S. agalactiae* were incubated for 18−24 h at 37°C, while *N. gonorrhoeae* incubated anaerobically for 48 h at 37°C. The diameter of the inhibition zones surrounding each well was measured [29]. All tests were performed in triplicate.

### Heavy Metal Detection of *A. paniculata* Intimate Wash

Heavy metals such as arsenic (As), cadmium (Cd), plumbum (Pb), and mercury (Hg) in the *A. paniculata* intimate wash were analyzed using a PinAAcle 900Z Atomic Absorption Spectrometer (AAS) (PerkinElmer, USA). In this process, 1 mL of final working concentration of *A. paniculata* intimate wash was mixed with 5 mL of concentrated nitric acid and 0.5 mL of 30% hydrogen peroxide in a digestion vessel. The mixture was digested at 200°C for 30 min in a microwave digester until complete digestion was achieved. It was then cooled to 70°C, transferred into a 25 mL volumetric flask, filtered through filter paper, and diluted to 25 mL with deionized water. The prepared solution was then analyzed using AAS, with a detection and quantification range of 0.01–1.875 mg/kg [30].

### Cytotoxicity Testing of the *A. paniculata* Extract using Brine Shrimp Lethality Assay

For cytotoxicity testing, *A. paniculata* extract was used instead of *A. paniculata* intimate wash formulation. The intimate wash formulation contains surfactants, preservatives, solubilizing agents, and pH adjusters, which may independently cause mortality in brine shrimp larvae and do not reflect the safety profile of the herbal extract itself [31].

Three milliliters of *A. paniculata* extract in 1% of DMSO (10 mg/mL) was serially diluted with sterile distilled water to a final concentration of 0.01953125 mg/mL. This concentration range was determined from preliminary screening results and previously published brine shrimp lethality assay protocols, ensuring that the selected doses covered both minimal and substantial mortality effects to enable precise calculation of the lethal concentration of 50% (LC_50_) value [32].

Seawater was prepared by dissolving 38 g of non-iodized sea salt in 1 L of distilled water and the pH was adjusted to 8.5 with sodium hydroxide. Then, 1 g of brine shrimp cysts (with 95% hatching rate) was incubated in the prepared seawater for 24 h under continuous illumination. For the assay, 500 μL of diluted extract and 4.5 mL seawater were added to Falcon tubes containing ten nauplii, with the control containing 500 μL of 1% DMSO and 4.5 mL seawater. Each concentration was tested in triplicate, and mortality was recorded after 24 h [33]. Percentage mortality was calculated using the following formula, and the LC_50_ was determined by plotting mortality percentage against the concentration of the extract [34].

$$\text{Percentage of mortality=}\frac{\text{No. of dead nauplii}}{\text{Total no. of nauplii}}\times 100$$

### Stability Study of the *A. paniculata* Intimate Wash

According to National Pharmaceutical Regulatory Agency (NPRA) real-time testing guidelines, the manufactured *A. paniculata* intimate wash was required to be stored at a temperature range of 30°C ± 2°C with a relative humidity of 75% ± 5% [35]. The stability study was conducted right after the preparation, followed by every three months for the first year, and then every six months for the second year. The formulation was evaluated for its physical characteristics only before and after the storage. The assessed physical attributes included colour, odour, pH, and texture. These parameters were observed by naked eyes.

Instrumental analyses such as colorimeter, texture analyser, or high-performance liquid chromatography (HPLC) quantification of andrographolide were not performed, as the present study focused solely on preliminary physical stability observations during storage. Therefore, the findings were not interpreted as evidence of chemical stability of the active compounds.

### Sterility Testing of the *A. paniculata* Intimate Wash

Nutrient Agar and Potato Dextrose Agar plates were labelled and divided into four quadrants, each representing *A. paniculata* intimate wash concentrations of 0.1%, 0.5%, 1.0%, and 5.0% (v/v). Prior to sterility testing, neutralization of residual antimicrobial activity at each formulation concentration was validated using Dey-Engley Neutralizing Broth as described above. Subsequently, 10 μL of each formulation was dispensed onto the corresponding quadrant and spread evenly over the agar surface. The inoculated plates were allowed to absorb completely before incubation at 37°C for 24 h. Each concentration was tested in triplicate. Following incubation, the plates were examined for microbial growth. Where microbial growth was observed, viable colonies were enumerated and expressed as CFU/mL using the following formula [36].

$$\text{Viable bacterial count}\left(\frac{CFU}{ml}\right)=\left(\frac{\text{Number of colonies}}{\text{Total dilution factor}\times \text{Volume of culture plated in ml}}\right)$$

### Data Analysis

Statistical analysis was conducted using Statistical Package for the Social Sciences (SPSS) software version 29.0.1 (IBM Inc., USA). All experiments for the agar well diffusion method and brine shrimp lethality assay were performed in triplicate (n = 3). Data obtained from the agar well diffusion assay were analysed using one-way ANOVA followed by Bonferroni post-hoc analysis to determine significant differences between the test groups, PC and NC group, with *P* < 0.05 considered statistically significant [37]. The Bonferroni post-hoc test was selected to account for multiple pairwise comparisons and control the family-wise Type I error rate, thereby reducing the risk of false-positive findings. Data from the brine shrimp lethality assay were analysed using probit analysis to determine the LC_50_ values at 95% confidence interval, also with *P* < 0.05 regarded as statistically significant [38]. The mean and standard deviation of pH values were also calculated using SPSS. All data were recorded in Microsoft Excel (Microsoft Corp., USA).

## Results

### Neutralization Validation

Neutralization validation demonstrated that the neutralizing procedure effectively eliminated residual antimicrobial activity of the *A. paniculata* intimate wash formulation at all concentrations evaluated (0.1 to 5.0%, v/v). Growth inhibition was observed in samples exposed to the formulation without neutralization, confirming the antimicrobial activity of the formulation components. In contrast, microbial recovery was restored following treatment with Dey-Engley Neutralizing Broth.

Microbial growth observed in the neutralizer toxicity control was comparable to the viability control, indicating that the neutralizing broth did not exert inhibitory effects on *E. coli*. These findings demonstrated that Dey-Engley Neutralizing Broth effectively neutralized the residual antimicrobial activity of the intimate wash formulation across all concentrations tested and was suitable for microbial recovery during sterility assessment.

### Antimicrobial Activity of *A. paniculata* Intimate Wash

All five concentrations of *A. paniculata* intimate wash; 50, 25, 12.5, 6.25 and 3.125 mg/mL were tested against *C. albicans*, *N. gonorrhoeae*, *E. coli*, and *S. agalactiae*. SebaMed^®^ Feminine Wash and sterile distilled water served as PC and NC, respectively (Fig. 1). The zones of inhibition indicated that all concentrations of *A. paniculata* intimate wash exhibited statistically significant differences (*P* < 0.05) compared to the NC against all tested vaginal pathogens (Table 1).

The broth microdilution assay was previously performed following the standard microbiological procedures described in the study by Razian *et al*. [20] to determine the minimum inhibitory concentration (MIC) and minimum bactericidal concentration (MBC). The published study reported MIC and MBC values of 3.125 mg/mL for *C. albicans* and *E. coli*, while *S. agalactiae* exhibited an MIC below the lowest concentration tested and an MBC of 0.0781 mg/mL (Table 2) [20]. These findings complement the agar well diffusion assay by confirming antimicrobial activity under broth conditions, where antimicrobial efficacy is evaluated independently of compound diffusion through the agar matrix. The MIC and MBC data for *C. albicans*, *E. coli*, and *S. agalactiae* were reproduced from Razian *et al*. [20], while the MIC and MBC data for *N. gonorrhoeae* were obtained from previously generated experimental data using the same broth microdilution assay. *N. gonorrhoeae* exhibited an MIC below the lowest concentration tested (<0.0015 mg/mL) and an MBC of 0.078 mg/mL, which were identical to the values observed for *S. agalactiae*.

For *C. albicans*, *A. paniculata* intimate wash demonstrated moderate inhibition from 18.58 ± 0.39 mm to 21.77 ± 0.46 mm. The highest inhibition zone was recorded at 6.25 mg/mL (21.77 ± 0.46 mm) and the inhibition zones were relatively similar across all tested concentrations of the intimate wash. Statistical analysis showed that only the concentrations of 50 mg/mL (18.58 ± 0.39 mm), 25 mg/mL (18.98 ± 0.75 mm), and 6.25 mg/mL (21.77 ± 0.46 mm) produced significantly different inhibition zones compared to PC, which showed a high antimicrobial effect (20.35 ± 0.38 mm). The *A. paniculata* intimate wash produced inhibition zones of 21.77 ± 0.46 mm at 6.25 mg/mL and 21.33 ± 0.50 mm at 3.125 mg/mL. The inhibition zone at 6.25 mg/mL was significantly higher than that of the PC, whereas the difference observed at 3.125 mg/mL was not statistically significant.

For *N. gonorrhoeae*, the highest inhibition zone was observed at 6.25 mg/mL (28.08 ± 0.95 mm). Other concentrations of *A. paniculata* intimate wash exhibited concentration-dependent inhibition trends, with only slight differences in inhibition zones (24.58 ± 0.74 mm to 27.73 ± 0.39 mm). However, no significant differences were observed compared to PC. Compared to the PC (26.75 ± 0.80 mm), the *A. paniculata* intimate wash demonstrated notable inhibition against *N. gonorrhoeae*, with some tested concentrations producing slightly larger inhibition zones than the PC.

The antimicrobial activity of *A. paniculata* intimate wash against *E. coli* remained almost the same for all tested concentrations with zones ranging from 9.53 ± 0.47 to 10.22 ± 1.16 mm. The highest zone inhibition was observed at 6.25 mg/mL (10.22 ± 1.16 mm). No significant differences were detected compared to PC. The *A. paniculata* intimate wash produced inhibition zones against *E. coli* across all tested concentrations, with inhibition-zone values observed within the range of those produced by the PC. These findings indicate that the formulation exhibited antimicrobial activity against *E. coli*.

For *S. agalactiae*, the *A. paniculata* intimate wash produced inhibition zones ranging from 11.55 ± 0.85 to 13.30 ± 0.79 mm across the tested concentrations. The highest inhibition was observed at 50 mg/mL (13.30 ± 0.79 mm), with the inhibition zone exceeding that of the PC (13.18 ± 0.46 mm). This suggests that the *A. paniculata* intimate wash has promising antimicrobial activity against *S. agalactiae*.

The *A. paniculata* intimate wash demonstrated antimicrobial activity against all tested pathogens. The PC showed strong inhibition against all tested pathogens, whereas no inhibition zones were observed for the NC. Across the tested concentration range (3.125 to 50 mg/mL), the inhibition zones produced by the *A. paniculata* intimate wash showed minimal variation among concentrations. The lowest tested concentration (3.125 mg/mL) produced inhibition zones against all tested pathogens, with inhibition zones that were generally similar to those observed at higher concentrations. Based on these findings, 3.125 mg/mL was selected as the working concentration for subsequent toxicity and stability evaluations.

### Heavy Metals Analysis of *A. paniculata* Intimate Wash

Analysis of heavy metal levels showed that all tested metals were below the permissible limits specified in the Poison Act 1952 guidelines [39] for herbal products and were therefore considered not detected (Table 3). The permissible detection limits for the heavy metals, arranged in descending order, were Pb (2.50 mg/kg), As (1.875 mg/kg), Cd (0.15 mg/kg), and Hg (0.01 mg/kg). These results indicated that the *A. paniculata* intimate wash contains heavy metal levels that fall within the safe limits.

### Cytotoxicity of *A. paniculata* Extract

The results of the brine shrimp lethality assay, together with the LC_50_ value, are presented in Fig. 2. The mortality curve showed a concentration-dependent trend and LC_50_ value was calculated to be at 7.25 mg/mL with the confidence interval at 4.83 to 13.03 mg/mL. The higher the concentration of *A. paniculata* extract used, the higher the percentage of mortality.

According to Table 4, at the lowest concentrations ranging from 0.3125 to 1.25 mg/mL, mortality was minimal at 3.33%. A sharp increase was observed at 2.5 mg/mL (40%) and 5 mg/mL (43.33%), while the maximum mortality of 50% occurred at 10 mg/mL. No mortality was observed at *A. paniculata* extract concentrations between 0.01953125 mg/mL and 0.15625 mg/mL. No mortality was also observed in the control tube using seawater and DMSO.

The concentrations used in the brine shrimp lethality assay were lower than those evaluated in the antimicrobial assay. Preliminary antimicrobial screening indicated that these lower concentrations did not produce significant inhibitory effects against the tested pathogens.

### Stability Study of *A. paniculata* Intimate Wash

Formulated *A. paniculata* intimate wash was stored at 30°C ± 2°C under 75% ± 5% relative humidity in accordance with NPRA guidelines for real-time stability testing. These conditions are representative of hot and humid tropical climates, as recognized by NPRA to stimulate prolonged storage effects at room temperature. The results in Table 5 showed that the intimate wash formulation remained almost stable after the time interval of storage; 3, 6, 9, 12, 18 and 24 months.

The total visible change was observed in colour at the nine-month interval compared to freshly prepared intimate wash. Nevertheless, the intimate wash remained clear without turbidity, and its texture was maintained throughout the storage period, indicating its strong stability.

Other evaluated physical characteristics include odour and pH of *A. paniculata* intimate wash. All formulated intimate wash retained the strong odour of peppermint for up to nine months. For the pH evaluation, no substantial changes were observed during storage, with recorded values ranging from 3.70 ± 0.00 to 3.86 ± 0.12 at preliminary, 3, 6, 9, 12, 18, and 24 months. The relatively stable pH values suggest that the formulation maintained acceptable physical stability under the tested storage conditions. However, no chemical analyses were performed to confirm the stability of active compounds during storage.

### Sterility Study of *A. paniculata* Intimate Wash

To ensure the quality and safety of the formulated intimate wash, microbiological assessment was conducted. The intimate wash was tested from the day of freshly prepared, till the time intervals; 3, 6, 9, 12, 18 and 24 months.

Fig. 3 showed that there was no contamination of microorganisms observed in the formulated *A. paniculata* intimate wash during the storage. There was no growth of bacteria or fungi on Nutrient Agar or Potato Dextrose Agar after testing with 0.1%, 0.5%, 1.0%, and 5.0% (v/v) *A. paniculata* intimate wash concentrations.

## Discussion

This study demonstrated that *A. paniculata* intimate wash exhibited antimicrobial activity against selected vaginal pathogens. Antimicrobial activity was demonstrated using agar well diffusion assays for all tested pathogens, while broth microdilution data provided complementary MIC and MBC findings for *C. albicans*, *E. coli*, and *S. agalactiae*, which were reproduced from Razian *et al*. [20], and for *N. gonorrhoeae*, which were obtained from previously generated experimental data. The formulation produced measurable inhibition zones against all tested pathogens at the lowest concentration evaluated (3.125 mg/mL), which was subsequently selected as the final working concentration for further toxicity and stability evaluations [40]. Therefore, antimicrobial potency against this pathogen was interpreted solely from agar well diffusion results.

The relatively small differences in inhibition zone diameters across the tested concentration range may be attributed to the concentrations evaluated being at or above the inhibitory threshold for some microorganisms. Based on the broth microdilution results, the tested concentrations in the agar well diffusion assay included the MIC values for *C. albicans* and *E. coli*, while *N. gonorrhoeae* and *S. agalactiae* exhibited an MIC below the lowest concentration tested (<0.0015 mg/mL). Therefore, further increases in formulation concentration may not have resulted in proportional increases in inhibition zone diameters because antimicrobial activity may have already approached a maximum effect within the tested range. In addition, diffusion characteristics of antimicrobial compounds through the agar matrix may have contributed to the limited expansion of inhibition zones. Thus, the observed plateau effect is likely influenced by both the antimicrobial response reaching a saturation point within the tested concentration range and the inherent diffusion limitations of the agar-based assay.

For *C. albicans*, an inverse trend was observed, whereby the inhibition zone diameter increased as the concentration of *A. paniculata* intimate wash decreased. This unexpected pattern should not be interpreted as evidence of greater antifungal potency at lower concentrations. Rather, the observed variation may be related to differences in the diffusion of antimicrobial constituents through the agar matrix at different formulation concentrations [41]. Since agar well diffusion results depend on both antimicrobial activity and the ability of the extract to diffuse through the agar, inhibition zone diameter may not always increase proportionally with increasing concentration. The MIC of 3.125 mg/mL obtained from the broth microdilution assay provides complementary evidence of the inhibitory concentration under liquid-phase conditions [40]. Therefore, the inverse concentration–inhibition zone relationship observed for *C. albicans* should be interpreted cautiously, particularly because inhibition zone diameter alone does not directly quantify antifungal potency [42].

The antimicrobial activity of the *A. paniculata* intimate wash may be associated with bioactive constituents such as andrographolide. However, as andrographolide content was not quantified in the present study, its specific contribution could not be confirmed. Other formulation components, including peppermint oil and excipients, may also have contributed to the observed antimicrobial activity [20]. Peppermint oil, which was incorporated at a concentration of 2% in the formulation, is present below concentrations previously reported to exhibit antimicrobial activity and is therefore unlikely to contribute substantially to the overall antimicrobial effect [43, 44]. The observed antimicrobial effects may also involve additive or potentially synergistic interactions between bioactive compounds, preservatives, and surfactants, which could enhance membrane permeability and antimicrobial penetration [45, 46].

Safety evaluation showed that heavy metal content in the formulation was within permissible limits established by NPRA Malaysia [39]. These findings are consistent with previous reports indicating low heavy metal accumulation in *A. paniculata* and confirming its safety when within WHO permissible limits [47, 48]. However, plant materials may still accumulate trace metals depending on environmental conditions, highlighting the importance of quality control [49]. In addition, exposure to heavy metals may influence phytochemical biosynthesis, including andrographolide levels, indicating environmental sensitivity of bioactive compounds [50].

Cytotoxicity evaluation assay showed that *A. paniculata* extract was non-toxic onto brine shrimp, with LC_50_ values exceeding 1 mg/mL, consistent with Meyer’s toxicity index [51, 52]. However, surfactant-containing formulations were not tested due to possible interference in the assay, as surfactants may disrupt membranes and artificially increase mortality rates [31]. Although a previous study reported non-toxic effects of formulations containing similar surfactants [51], discrepancies suggest that formulation composition may influence toxicity outcomes. Therefore, further studies using relevant mammalian cell models are required.

In this study, Sebamed^®^ Feminine Intimate Wash, the commercial comparator, was included only as a reference product in the antimicrobial assays. However, these claims do not provide quantitative cytotoxicity data from standardized *in vitro* assays. Therefore, direct comparison of the cellular safety profile between the developed formulation and the commercial product could not be established. According to the manufacturer, the product has been dermatologically, gynecologically, and clinically tested and is formulated at pH 3.8 to support vaginal microflora balance and maintain the natural acidic environment, suggesting good tolerability under routine use [53].

However, these claims do not provide quantitative cytotoxicity data from standardized *in vitro* assays such as 3-(4,5-dimethylthiazol-2-yl)-2,5-diphenyltetrazolium bromide assay, lactate dehydrogenase assay, or neutral red uptake assays, and peer-reviewed evidence on its cytotoxicity toward mammalian cell lines remains limited. Thus, future studies should include cytotoxicity assessment of both the developed formulation and commercial intimate wash products to enable a more comprehensive and evidence-based safety comparison.

Toxicity evaluation remains essential for vaginal applications, as current safety data for *A. paniculata*-based intimate wash formulations are limited. The present study provides only preliminary toxicity information, and further testing using vaginal epithelial cell lines such as VK2/E6E7 is recommended to better assess mucosal safety [54]. Ultimately, clinical studies are required to confirm safety and efficacy under real-use conditions [55].

Stability testing over 24 months showed that the formulation remained physically and microbiologically stable under controlled conditions (30 ± 2°C and 75 ± 5% relative humidity). Minor changes in colour from greenish to light amber were observed, which may reflect normal physical changes in herbal-based formulations during long-term storage. However, no chemical analysis was performed. Therefore, the underlying chemical changes responsible for this colour shift could not be confirmed [56, 57]. The gradual loss of peppermint aroma may be attributed to volatility and degradation of menthol compounds over time [58, 59]. Despite these changes, the formulation maintained an acidic pH suitable for vaginal use due to citric acid buffering [60]. No precipitation or microbial contamination was detected, likely due to preservatives such as sodium benzoate and potassium sorbate, which effectively inhibit microbial growth [61].

Overall, this study demonstrates that the *A. paniculata* intimate wash possesses promising antimicrobial activity and good stability, while the *A. paniculata* crude extract exhibited acceptable preliminary safety in the cytotoxicity study, supporting the formulation's potential as a natural alternative for feminine hygiene. However, further *in vitro* and clinical investigations are necessary to confirm its safety, efficacy, and microbiome compatibility. Evaluation against beneficial vaginal microbiota such as L. crispatus is also recommended to ensure microbiome compatibility. Additionally, expanding heavy metal analysis to include elements such as chromium, iron, and nickel would provide a more comprehensive safety assessment of the formulation.

## Supplemental Materials

Supplementary data for this paper are available on-line only at http://jmb.or.kr.



## Figures and Tables

**Fig. 1:**
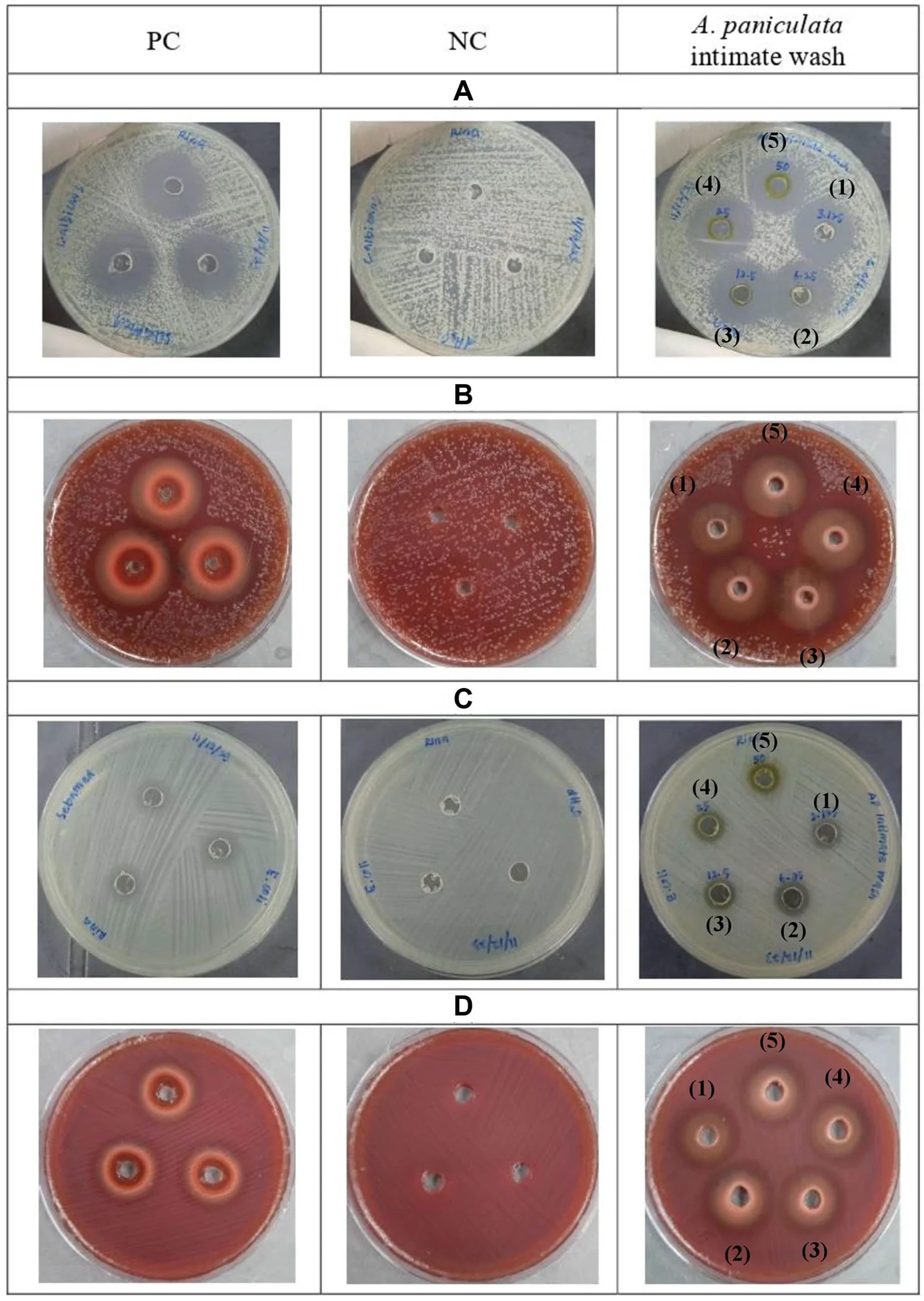
Antimicrobial activity of different concentrations of *A. paniculata* intimate wash. Note: (1) 3.125 mg/mL, (2) 6.25 mg/mL, (3) 12.5 mg/mL, (4) 25 mg/mL, (5) 50 mg/mL

**Fig. 2:**
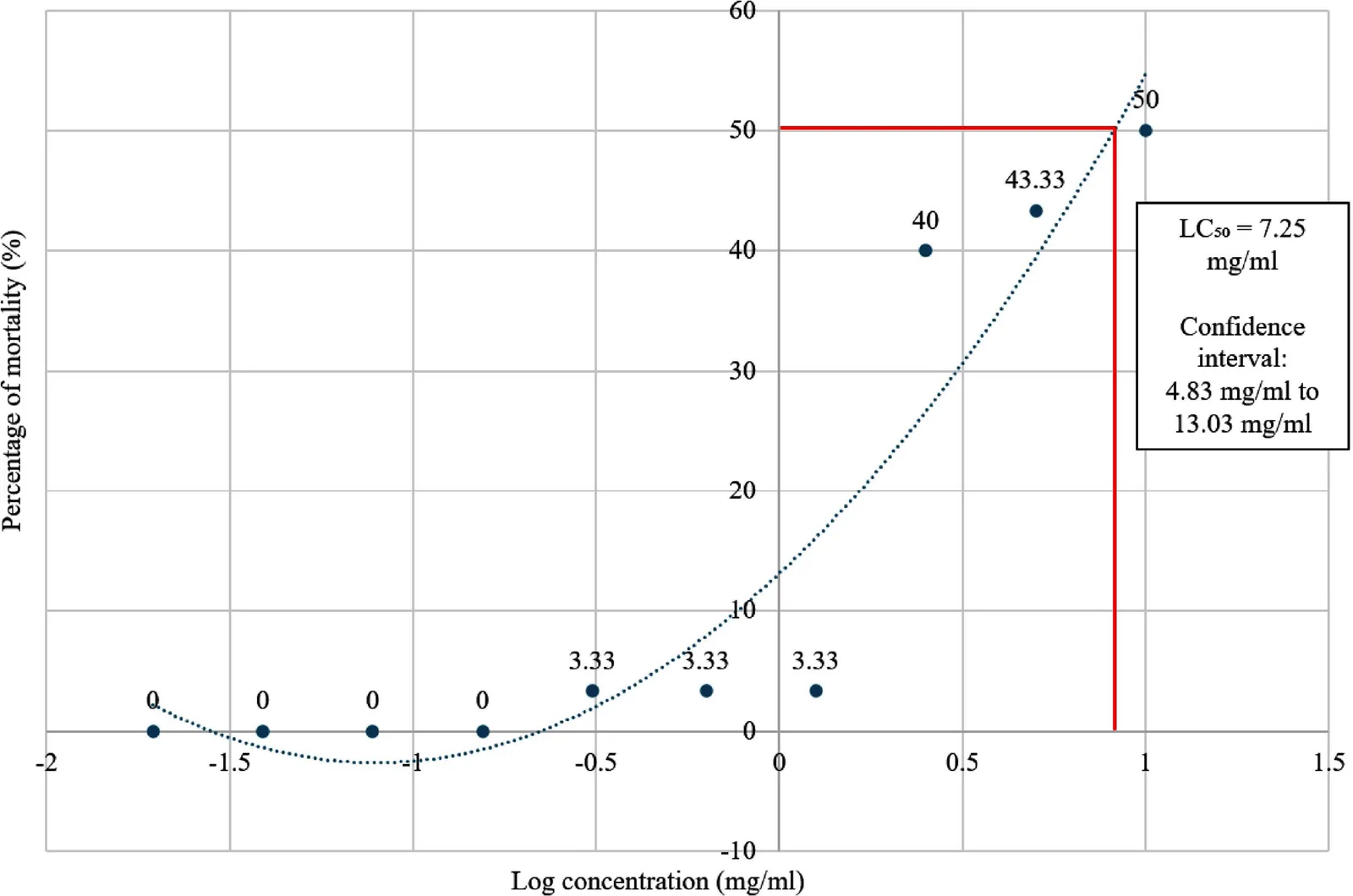
Determination of LC_50_ value for *A. paniculata* extract using brine shrimp lethality assay.

**Fig. 3:**
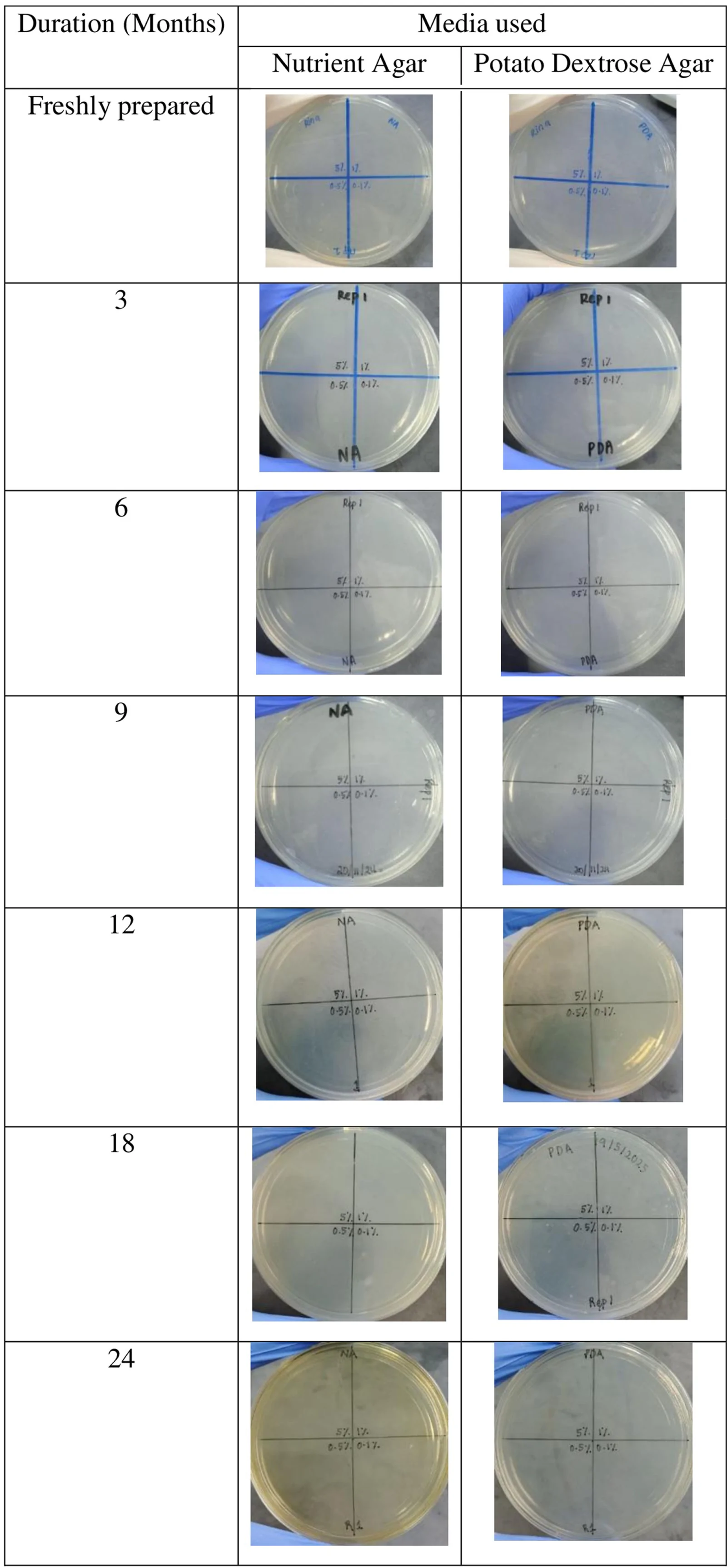
Microbiological contamination assessment of *A. paniculata* intimate wash after the time interval storage. Note: The *A. paniculata* intimate wash concentrations used are 0.1%, 0.5%, 1.0% and 5.0% (v/v) as labelled on the plate.

**Table 1:** Diameter zone inhibitions (mm) of *A. paniculata* intimate wash against selected vaginal pathogens using agar well diffusion method.

Tested pathogens	PC	NC	*A. paniculata* intimate wash concentration (mg/mL)				
	SebaMed^®^ Feminine Wash	Sterile distilled water	50	25	12.5	6.25	3.125
*C. albicans*	20.35 ± 0.38	0.00 ± 0.00	18.58 ± 0.39^#*^	18.98 ± 0.75^#*^	20.27 ± 0.75^*^	21.77 ± 0.46^#*^	21.33 ± 0.50^*^
*N. gonorrhoeae*	26.75 ± 0.80	0.00 ± 0.00	27.05 ± 0.92^*^	27.65 ± 0.42^*^	27.73 ± 0.39^*^	28.08 ± 0.95^*^	24.58 ± 0.74^*^
*E. coli*	10.48 ± 1.47	0.00 ± 0.00	10.03 ± 0.52^*^	9.75 ± 0.66^*^	9.63 ± 0.42^*^	10.22 ± 1.16^*^	9.53 ± 0.47^*^
*S. agalactiae*	13.18 ± 0.46	0.00 ± 0.00	13.30 ± 0.79^*^	11.55 ± 0.85^*^	12.15 ± 0.74^*^	12.15 ± 0.71^*^	11.58 ± 1.40^*^

Data are expressed as mean ± standard deviation. ^#^ denotes a statistically significant difference (*P* < 0.05) compared with the positive control (PC), while ^*^ denotes a statistically significant difference (*P* < 0.05) compared with the negative control (NC).

Note: No inhibition zones of all tested pathogens for NC.

**Table 2:** MIC and MBC of the *A. paniculata* intimate wash against selected vaginal pathogens.

Pathogens	MIC (mg/mL)	MBC (mg/mL)
*C. albicans*	3.125	3.125
*N. gonorrhoeae*	<0.0015	0.078
*E. coli*	3.125	3.125
*S. agalactiae*	<0.0015	0.078

#This table is adapted from Razian *et al*. [20] under the terms of the Creative Commons Attribution License. Data for *C. albicans*, *E. coli* and *S. agalactiae* were reproduced from Razian *et al*. [20], whereas MIC and MBC data for *N. gonorrhoeae* were generated in the present study using the broth microdilution assay.

Note: Minimum inhibitory concentration (MIC) values reported as < 0.0015 mg/mL indicate that growth inhibition occurred at a concentration below the lowest concentration tested in the broth microdilution assay. Minimum bactericidal concentration (MBC) values represent the lowest concentration that resulted in microbial killing after subculture.

**Table 3:** Heavy metals analysis by graphite furnace absorption spectrometry (GFAAS) and flow injection mercury system (FIMS) in *A. paniculata* intimate wash.

Test parameter	Test method	Poison Act 1952 (mg/kg)	LOD (mg/kg)	Results (mg/kg)
Arsenic (As)	GFAAS	5.00	1.875	ND
Cadmium (Cd)	GFAAS	0.30	0.15	ND
Plumbum (Pb)	GFAAS	10.00	2.50	ND
Mercury (Hg)	FIMS	0.50	0.01	ND

*ND is not detected, LOD is limit of detection

**Table 4:** Percentage of mortality of brine shrimp after *A. paniculata* extract treatment.

Concentration (mg/mL)	Mean of mortality	Percentage of mortality (%)	Log concentration	Probit
Control	0.0000	0.00	0.00	0.00
10	0.5000	50.00	1.00	5.00
5	0.4333	43.33	0.70	4.82
2.5	0.4000	40.00	0.40	4.75
1.25	0.0333	3.33	0.10	3.12
0.625	0.0333	3.33	-0.20	3.12
0.3125	0.0333	3.33	-0.51	3.12
0.15625	0.0000	0.00	-0.81	0.00
0.078125	0.0000	0.00	-1.11	0.00
0.0390625	0.0000	0.00	-1.41	0.00
0.01953125	0.0000	0.00	-1.71	0.00

Note: Total brine shrimp (n) = 10 nauplii

Note: Lower concentrations used in the toxicity assay were not included in the antimicrobial assay because preliminary screening showed no significant antimicrobial activity at these concentrations.

**Table 5:** The characterization of *A. paniculata* intimate wash after the time interval storage.

Parameter	Freshly prepared	3 Months	6 Months	9 Months	12 Months	18 Months	24 Months
Colour	Originally greenish	Remained greenish	Slightly greenish after shaking	Shifted to amber	Light amber colour	Light amber colour	Light amber colour
Odour	Fresh peppermint scent	Remained peppermint scent	Remained peppermint scent	Remained peppermint scent	Peppermint scent was slowly degraded	Still has slight peppermint scent	Still has slight peppermint scent
pH	3.70 ± 0.00	3.76 ± 0.28	3.71 ± 0.12	3.79 ± 0.16	3.82 ± 0.16	3.86 ± 0.12	3.79 ± 0.18
Texture	Clear; no visible particles	Remained clear	Remained clear	Remained clear	Remained clear	Remained clear	Remained clear
